# Development of Novel Herbal Compound Formulations Targeting Neuroinflammation: Network Pharmacology, Molecular Docking, and Experimental Verification

**DOI:** 10.1155/2023/2558415

**Published:** 2023-05-24

**Authors:** Yang Liu, Dennis Chang, Xian Zhou

**Affiliations:** NICM Health Research Institute, Western Sydney University, Westmead, NSW 2145, Australia

## Abstract

Neuroinflammation plays an important role in the onset and progression of neurodegenerative diseases. The multicomponent and multitarget approach may provide a practical strategy to address the complex pathological mechanisms of neuroinflammation. This study aimed to develop synergistic herbal compound formulas to attenuate neuroinflammation using integrated network pharmacology, molecular docking, and experimental bioassays. Eight phytochemicals with anti-neuroinflammatory potential were selected in the present study. A compound-gene target-signaling pathway network was constructed to illustrate the mechanisms of action of each phytochemical and the interactions among them at the molecular level. Molecular docking was performed to verify the binding affinity of each phytochemical and its key gene targets. An experimental study was conducted to identify synergistic interactions among the eight phytochemicals, and the associated molecular mechanisms were examined by immunoblotting based on the findings from the network pharmacology analysis. Two paired combinations, andrographolide and 6-shogaol (AN-SG) (IC_50_ = 2.85 *μ*g/mL), and baicalein-6-shogaol (BA-SG) (IC_50_ = 3.28 *μ*g/mL), were found to synergistically (combination index <1) inhibit the lipopolysaccharides (LPS)-induced nitric oxide production in microglia N11 cells. Network pharmacology analysis suggested that MAPK14, MAPK8, and NOS3 were the top three relevant gene targets for the three phytochemicals, and molecular docking demonstrated strong binding affinities of the phytochemicals to their coded proteins. Immunoblotting suggested that the AN-SG and BA-SG both showed prominent effects in inhibiting inducible nitric oxide synthase (iNOS) (*p* < 0.01 and *p* < 0.05, respectively) and MAPKp-p38 (both *p* < 0.05) compared with those induced by the LPS stimulation only. The AN-SG combination exhibited greater inhibitions of the protein expressions of iNOS (*p* < 0.05* vs*. individual components), which may partly explain the mechanisms of the synergy observed. This study established a practical approach to developing novel herbal-compound formulations using integrated network pharmacology analysis, molecular docking, and experimental bioassays. The study provides a scientific basis and new insight into the two synergistic combinations against neuroinflammation.

## 1. Introduction

Neurodegenerative diseases are characterised by the progressive loss of neurons and the deposition of proteins manifested as altered physicochemical properties in the brain and peripheral organs [1]. It is a group of major diseases in the elderly that significantly impacts families, communities, and healthcare systems worldwide. The etiologic and underlying pathophysiology of neurodegenerative diseases is complex and mediated by various factors [2]. To date, there is no treatment available to prevent or cure neurodegenerative diseases due to the lack of understanding of the causes and pathological mechanisms [3–5].

Neuroinflammation refers to the activation of the brain's innate immune system and the abnormal secretion of proinflammatory cytokines in response to an inflammatory challenge [6]. Emerging evidence has shown that neuroinflammation is associated with the onset and development of many neurodegenerative diseases, such as Alzheimer's disease, Parkinson's disease, and amyotrophic lateral sclerosis [7]. Microglia are the resident immune cells in the central nervous system that play a crucial role in normal brain function and neuroinflammation-mediated neuronal pathology [8]. Under the pathological conditions, the excessive activation of microglia triggers the elevated production of proinflammatory mediators [e.g., nitric oxide (NO) and tumour necrosis factor (TNF)-*α*] that lead to the high permeability of the blood-brain barrier (BBB) and impaired neuronal survival [9]. Thereby, neuroinflammation has been implicated as an important therapeutic target for neurodegenerative diseases [10, 11].

There has been increasing interest in studying phytochemicals for their potential anti-neuroinflammatory effects [12, 13]. In the current study, a literature review was conducted to identify phytochemicals that possess anti-neuroinflammatory potential, and those that are commercially available were sourced for preliminary laboratory testing. Based on the results [14], eight phytochemicals were finally selected in the present study. Luteolin (LU), a flavonoid found in various vegetables, medicinal herbs and fruits, possesses anti-inflammatory, antioxidant, neuroprotective, neurotrophic, and neurogenesis activities [15]. Baicalein (BA), a main bioactive ingredient from the root of *Scutellaria baicalensis* Georgi, exhibits potent anti-neuroinflammatory and neuroprotective effects [16]. Andrographolide (AN) is a major active constituent of *Andrographis paniculate* Burm. f., that has been shown to have potent antioxidant and anti-neuroinflammatory properties [17, 18]. 6-shogaol (6-SG), a bioactive ingredient in dried ginger, possesses a strong anti-neuroinflammatory property [19–21] and has been shown to improve memory function in animal models of cognitive disorders [20]. Curcumin (CU), the major bioactive component from *Curcuma longa* L., exerts broad and potent anti-inflammatory and anti-cytokine activities [22]. Hesperidin (HES) is a flavanoglycone abundantly present in citrus fruits, which exerts neuroprotective effects against PD and Huntington's disease by virtue of its antioxidant, anti-inflammatory, and anti-apoptotic actions [23]. Tetrandrine (TE) is a bis-benzylisoquinoline alkaloid that is extracted from the roots of *Stephania tetrandrae* S. Moore. TE possesses a diverse array of biological actions, including antineuroinflammatory and antioxidative activities [24, 25]. Glycyrrhizin (GLY), a triterpenoid saponin compound, is the main bioactive constituent of *Glycyrrhiza glabra* L., and has been shown to possess antineuroinflammatory and neuroprotective properties [26].

Combination therapy has become an emerging therapeutic strategy for complex diseases, such as neurodegenerative diseases, offering improved clinical outcomes and reduced toxicity through a multitarget approach [26–30]. The fundamental treatment principle of combination therapy is synergy, where the combined effects are greater than the sum of the individual effects [31]. Numerous studies have demonstrated synergistic or positive relationships among the bioactive compounds in complex drug combinations [31–35]. For instance, Park et al. [25] demonstrated that a combination of LU and L-theanine exhibited a greater effect in protecting hippocampus tissues than each compound used alone in an early-stage Alzheimer's disease animal model. Their results showed that LU-L-theanine attenuated memory impairment and prevented tau protein phosphorylation and norepinephrine depletion in rats infused with amyloid-*β* in the hippocampus [36]. Although there is generally a lack of rigorous mathematical tools to accurately interpret the interaction among bioactive compounds in herbal mixtures, various methodologies have been developed to help illustrate the individual action of active ingredients in an herbal formula. Network pharmacology is a computational and mathematical model integrating literature, experimental data, and the computational sciences [37]. Recently, it has been developed to demonstrate the multitargeted actions of components in combination therapy [38]. This method can identify the key bioactive compounds from a complex formulation, elucidate the gene targets associated with the disease, and build the network of the bioactive compounds, gene targets, and associated signaling pathways [39]. Its increasing popularity is attributed to low research costs, a short research cycle, and comprehensive information. In drug discovery investigations, there is an emerging trend of integrating analysed information from network pharmacology with experimental results to form novel combination therapy, with a known mechanism of action [40]. Network pharmacology is usually followed by molecular docking, which can determine the binding affinity between the key bioactive and associated protein target and partly validate the predicted mode of action of the bioactive compound from the network pharmacology analysis [40]. In addition, mathematical modelling, such as combination index (CI), is widely used to quantify the interaction of drug combinations (i.e., synergistic or antagonistic) on a specific biological target based on the experimental results [38, 41].

Although the individual anti-neuroinflammatory activity of the eight selected phytochemicals has been studied, there is generally a lack of a standard approach to form synergistic combination therapy, which leads to enhanced therapeutic outcomes. Based on the versatile pharmacological actions of these eight phytochemicals associated with neuroinflammation, it is plausible that these phytochemicals may exert synergistic interactions when used in combination, leading to improved pharmacological outcomes. This study aimed to establish an integrative approach for developing synergistic combinations of phytochemicals using in silico network pharmacology, molecular docking, and bioassay-based validations. Our research may provide a scientific basis and establish a new framework for novel interventions against neuroinflammation and neurodegenerative diseases.

## 2. Materials and Methods

### 2.1. Gene Targets Associated with Neuroinflammation

The two-dimensional (2D) structures of the eight phytochemicals were retrieved from the PubChem database (https://pubchem.ncbi.nlm.nih.gov/). Spatial Data File (SDF) of 2D structures was uploaded to the PharmMapper database to generate potential gene targets. Human normalisation of the Universal Protein Resource (UniProt) (https://www.uniprot.org/) was searched to filter the obtained gene targets and normalise their names and organisms. The characters of the eight phytochemicals were obtained from the Traditional Chinese Medicine Systems Pharmacology Database and Analysis Platform (TCMSP, https://tcmspw.com/tcmsp.php). According to literature reports and pharmacokinetic parameters, the phytochemicals with oral bioavailability (OB) ≥ 30% reflect good absorption and slow metabolism after oral administration. The phytochemicals with a drug-likeness (DL) ≥ 0.18 were chemically suitable for drug development. The phytochemicals with the BBB ≥ − 0.3 reflect good permeability through the BBB [42]. Neuroinflammation-related gene targets were obtained from the Online Mendelian Inheritance in Man (OMIM) Database (https://www.omim.org/), the GeneCards Database (https://www.GeneCards.org/), and the DisGeNET Database (https://www.disgenet.org/home/). Duplicate targets were removed after collecting all the gene targets of neuroinflammation in the three databases. The Venn diagram was drawn to show the number of overlapping gene targets related to the disease and each phytochemical.

### 2.2. Protein-Protein Interaction Network Construction

The protein-protein interaction (PPI) network describes physical interactions among protein targets that are associated with the phytochemical candidates and neuroinflammation [43]. The STRING (Search Tool for the Retrieval of Interacting Genes/Proteins) database (https://string-db.org/) provides the relevant protein-protein associations, which analyses confidence scores for each of the protein connections with quantified reliability. Overlapping gene targets of each phytochemical were input to STRING to generate the PPI network [44]. The disconnected edges were hidden in the default setting in the network, and the required interaction score was set at 0.9 as a minimum to obtain the network of PPI.

### 2.3. Gene Ontology and Kyoto Encyclopaedia of Genes and Genomes Pathway Analysis

Based on the constructed PPI network of each phytochemical, the associated gene ontology (GO) and Kyoto Encyclopedia of Genes and Genomes (KEGG) pathways that represent gene product properties and signaling pathways were investigated. The Database for Annotation, Visualization, and Integrated Discovery (DAVID) is the online enrichment analysis database (https://david.ncifcrf.gov/home.jsp) applied to explore the relevant gene function annotation and pathway enrichment. The GO targets were searched through the DAVID database to elucidate the interaction between related gene targets of each phytochemical and their associated GO when targeting neuroinflammation [45]. Following that, the cross-GO targets were uploaded to DAVID to obtain associated biological pathways (BP), cellular content (CC), molecular function (MF), and KEGG pathways. In the present study, the top 20 targets in each function were selected and input to Bioinformatics (https://www.bioinformatics.com.cn/) to conduct the enrichment analysis of KEGG pathways and GO enrichment used across genes, and the terms with a *p* value less than 0.05 were filtered for the subsequent network construction [46].

### 2.4. The Construction of Compound-Gene Targets-Signaling Pathway Network

The compound-gene targets-signaling pathway network was constructed using Cytoscape (v.3.8.2, Institute for Systems Biology, US) [34]. The network was built to examine the relationships among the phytochemicals, their gene targets, and associated pathways in targeting neuroinflammation. The image of the network and associated statistics were exported from Cytoscape. The relevant parameters were obtained by the “analysis network” tool in Cytoscape, including “Degree,” “Betweenness-Centrality,” “Closeness Centrality,” and “Stress.” The most relevant gene targets and KEGG pathways were further filtered by selecting those “Degree” larger than 2× median values [47]. The importance of the gene/protein target will be ranked by the degree, which will be used for the following experimental verification.

### 2.5. Molecular Docking Simulation

In order to evaluate the credibility of the connection between the core protein targets with each phytochemical against neuroinflammation, CB-Dock (v.1.0, Yang Cao Lab, China) was used to perform molecular docking (https://clab.labshare.cn/cb-dock/php/blinddock.php). CB-Dock is a protein-ligand docking model designed to identify binding sites, analyse center and size, and conduct molecular docking [48]. It facilitates docking procedures and increases the accuracy of molecular docking. Cavity-focused docking increases the accuracy and hits ratio with blind docking [48].

Based on the network pharmacology analysis, the structures of the selected gene targets-coded proteins were obtained from Protein Data Bank (PDB). The crystal structures of each compound candidate were sourced from the PubChem database. The “spacefill” and “cartoon” parameters were set for the ligand and receptor, respectively. “Element” and “chain” were used for ligands and coloured receptors.

### 2.6. Cell Culture

Mouse microglia N11 (N11) cell line was kindly donated from Professor Gerald Münch, School of Medicine, Western Sydney University [49]. N11 cells were cultured in Dulbecco's Modified Eagle Medium (DMEM, Lonza, Australia) supplemented with 10% fetal bovine serum (Sigma–Aldrich, Australia) and 1% penicillin (Sigma–Aldrich, Australia). Cultured cells with over 90% confluency were digested with 0.25% trypsin (Thermo Fisher Scientific, Australia) for the following bioassays. Cells were passaged every two-three days until passage 30.

### 2.7. Preparation of Selected Phytochemicals and Lipopolysaccharides-Induced Neuroinflammation

Pure isolated phytochemicals LU, BA, AN, 6-SG, CU, HES, TE, and GLY (purity >98%), were purchased from Chengdu BioPurify (China). The identity and purity were confirmed by high-performance liquid chromatography (Supplementary Material 1). Each phytochemical was dissolved in dimethyl sulfoxide (DMSO) at a concentration of 100 mM. They were diluted with DMEM serum-free media before adding to the cells with a DMSO concentration of 0.1%. N11 cells were seeded in 96-well culture plates (Corning® Costar®, Sigma, Australia) at a density of 1 × 10^6^ cells/well. After the incubation of 24 h, the cells were treated with individual or paired combinations of the eight phytochemicals with a 1 : 1 ratio at various concentrations (0.23–82.29 *μ*g/mL) for 1 h prior to the stimulation of lipopolysaccharides (LPS) at 1 *μ*g/mL. The cells and cell supernatant were then subjected to the following bioassays after 24 h's LPS stimulation.

### 2.8. Nitric Oxide Assay

The cell supernatant (90 *μ*L) from each well was mixed with 90 *μ*L of Griess reagent [1% sulphanilamide in 5% phosphoric acid and N-(1-naphthyl)-ethylene diamine dihydrochloride] for the detection of nitrite production as an indicator of nitric oxide (NO) [50]. The production of NO was determined by measuring the optical density at 540 nm using a microplate reader (BMG Labtech Fluostar Optima, Mount Eliza, Victoria, Australia).

### 2.9. Alamar Blue Assay

Cell viability was evaluated 24 h after the LPS stimulation. After removing the supernatants, the cells were incubated with 100 *μ*L of Alamar Blue (0.01 mg/mL resazurin) [51]. The plate was then incubated for another 2 h in a humidified incubator at 37°C. The optical density of each well was measured from excitation of 545 nm and emission of 595 nm using a microplate reader (BMG Labtech Fluostar Optima, Mount Eliza, Victoria, Australia).

### 2.10. Western Blot Analysis

N11 cells were grown in T75 cell flasks (SARSTEDT, Australia) until confluence. Cells were then treated with individual AN, 6-SG, and BA at 25 *μ*M, and combinations of AN-SG [AN (25 *μ*M) + 6-SG (25 *μ*M), total concentration of 7.83 *μ*g/mL] and BA-SG [BA (25 *μ*M) + 6-SG (25 *μ*M), total concentration of 6.83 *μ*g/mL] or media with vehicle (0.1% DMSO) for 1 h before the activation of LPS (1 *μ*g/mL). After the incubation for 40 min or 24 h, cell pellets were harvested by centrifugation at 500 *g* for 5 min at 4°C. The cell pellets were mixed with radioimmunoprecipitation assay buffer (Santa Cruz Biotechnology, Australia) with 1% proteinase inhibitors (Cell Signaling Technologies, United States), and their concentrations were elucidated using Pierce™ BCA Protein Assay Kit (Thermo Fisher Scientific, Australia). The total proteins from each sample at 10 mg/mL were separated by the SDS-PAGE electrophoresis (PowerPac HC, BIORAD, Australia), and then the proteins were transferred to the PVDF membrane by the iBlot 2 gel transfer device (Thermo Fisher Scientific, Australia). The membranes were incubated with 3% bovine serum albumin (BSA, Scientifix, Australia) dissolved in PBST [PBS buffer plus 1% tween 20 (Thermo Fisher Scientific, Australia] for 1 h at room temperature. The membranes were incubated with primary antibodies against phospho-p38 MAPK (1 : 1000, cat. no. 4511), p38 MAPK (1 : 1000, cat. no. 8690), and iNOS (1 : 1000, cat. no. 13120) overnight at 4°C. GAPDH (1 : 1000, cat. no. 5174) was used as a loading control. The primary antibodies were probed with antirabbit HRP conjugated secondary antibodies (1 : 5000, cat. no. 7074) at room temperature for 2 h. All these antibodies were purchased from Cell Signaling Technology (United States). The immunoreactive bands on the membranes were incubated by the SuperSignal West Pico Plus ECL kit (Thermo Fisher Scientific, Australia) and visualised by the iBright CL750 (Thermo Fisher Scientific, Australia). Specific bands were analysed, and the intensity was quantified using Image*J* software.

### 2.11. Synergy Determination

The CI model was applied to determine the interaction of phytochemicals in the NO assay. The dose-response curves of single and combined phytochemicals on the NO assay were generated from the laboratory experiments. The data was then input to the CompuSyn software 2.0 (ComboSyn, Inc., USA) to generate the CI-fraction affected (Fa) curve, isobologram figure, and the CI values at all Fa values [52, 53]. In the CI-Fa curve, Fa refers to the default effect level of the combination set between 0 and 1. In our study, Fa presented suppressive responses on NO from 0% to 100%. The CI values were used to demonstrate the interaction, with CI < 1 representing synergistic interaction, CI = 1 representing no interaction (additive effect), and CI > 1 representing antagonistic interaction [30]. The isobologram graphics were used to show synergy at three set concentrations (Fa = 0.5, Fa = 0.75, and Fa = 0.9).

### 2.12. Statistical Analysis

Statistical analysis was conducted using GraphPad Prism 9.0 software (GraphPad Software Inc., USA). The data were shown as mean ± standard error of the mean (SEM) from at least three individual experiments. The relative IC_50_ values were determined from the constructed dose-response curves. The statistical comparison between groups was conducted by one-way ANOVA with the Tukey test, and *p* < 0.05 was considered statistically significant.

## 3. Results

### 3.1. Network Pharmacology Analysis of Selected Phytochemicals against Neuroinflammation

#### 3.1.1. Characteristic Molecular Actions of Eight Phytochemicals Targeting Neuroinflammation

**Figure 1:**
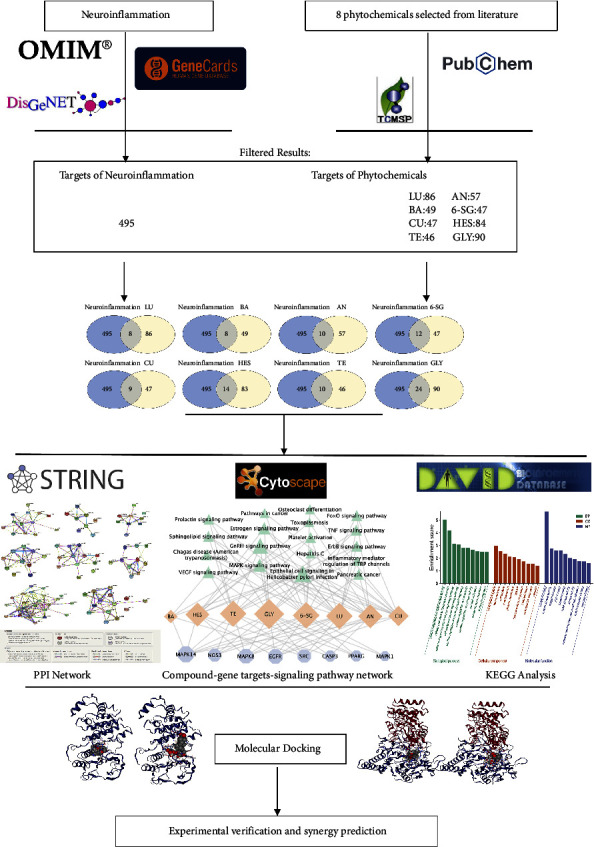
Workflow from network pharmacology analysis, molecular docking to experimental verification.

The compound-gene target-signaling pathway network was constructed to reveal the pharmacological action of each phytochemical in relation to neuroinflammation (Figure 1). Our results showed that 495 gene targets were associated with the pathology of neuroinflammation, and the top 100 gene targets were selected to check the intersection genes with each phytochemical. The Venn diagrams for the number of gene targets and intersections of gene targets for each phytochemical are shown in Supplementary Material 2. To check the drug-like property of selected phytochemicals, the screening criteria were defined as follows: BBB ≥ −0.3, DL ≥ 0.18, OB ≥ 30% [54]. The characteristics of each phytochemical, the number of total gene targets, and the intersections of genes with neuroinflammation are shown in Table 1. BA was suggested to have OB and DL parameters meeting the criteria and a high BBB permeability (BBB ≥ −0.3). Interestingly, although GLY did not show high OB and DL properties, it connects with the highest amount of total gene targets and intersections gene targets associated with neuroinflammation, suggesting its multitargeted (nonspecific) pharmacological action.

#### 3.1.2. PPI Network Construction and Analysis

The phytochemicals-disease PPI interaction network for each of the eight phytochemicals was created using the STRING database (Supplementary Material 3). In the PPI network, GLY displayed the most interactive PPI network, constructed by 24 nodes and 89 edges, with the average node degree at 7.42. It was followed by HES (nodes: 14 and edges: 32), LU (nodes: 13 and edges: 30), 6-SG (nodes: 12 and edges: 26), and AN (nodes: 10 and edges: 23). BA, CU, and TE showed 8, 9, and 10 nodes, respectively, and the average node degrees were all under 4.

#### 3.1.3. Analysis of GO Enrichment and KEGG Pathways

The GO and KEGG pathways analyses were conducted to investigate the associated protein targets and signaling pathways. A total of 202 GO entries were identified for all the phytochemicals. The top 10 significantly enriched terms for each phytochemical in the BP, MF, and CC categories are listed in Supplementary Material 4. Special attention was paid to the common BP observed among phytochemicals, where the crosstalk is likely to occur. For instance, the top common BPs of AN and 6-SG included peptidy-serine phosphorylation, sequence-specific DNA binding transcription factor activity, LPS-mediated signaling pathway, response to stress, and positive regulation of cyclase activity. The overlapping BPs for BA and 6-SG included positive regulation of cell growth and a LPS-mediated signaling pathway. The common CC was late endosome and caveola for AN and 6-SG and BA and 6-SG, respectively. There were several common MF involved in AN and 6-SG, including MAP kinase activity, ATP binding, protein serine/threonine kinase activity, protein binding, protein kinase activity, and protein phosphatase binding. However, BA and 6-SG only showed enzyme binding as the common MF.

The top 20 KEGG pathways of each phytochemical are shown in Supplementary Material 5. The KEGG pathways of LU, AN, 6-SG, HES, and GLY showed distinct patterns of distribution. TE only showed 11 KEGG pathways, and the pathways were at comparable degrees with no apparent distinction. CU and BA showed 9 and 3 KEGG pathways, respectively. The top common pathway among all eight phytochemicals was identified as the MAPK signaling pathway. In particular, the common KEGG pathway of AN and 6-SG was the MAPK signaling pathway, whereas the top KEGG pathway for BA and 6-SG was the VEGF signaling pathway. It was noticed that the MAPK signaling pathway had been shown to play a critical role in neuroinflammation [55].

#### 3.1.4. Construction of Compound-Gene Targets-Signaling Pathway Network

**Figure 2:**
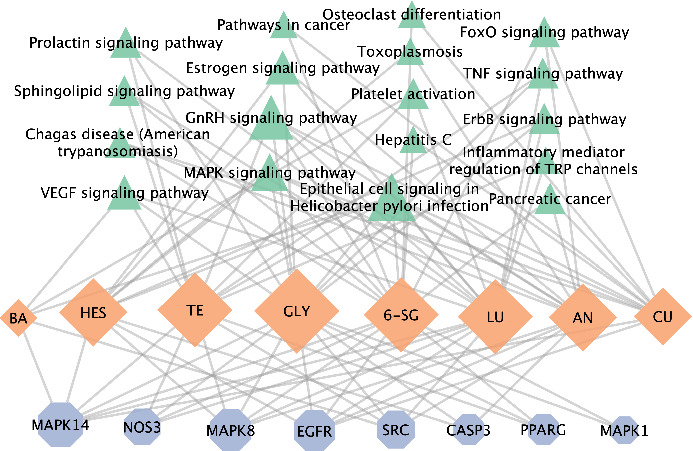
The compound-gene targets-signaling pathway network of the eight phytochemicals related to neuroinflammation. Orange nodes represent each phytochemical candidate, blue nodes refer to potential phytochemical's targets in neuroinflammation, and the green nodes display the signaling pathway. The size of each node represents its degree in the network. The grey connecting lines reflect that each node is interconnected.

The network of the eight phytochemicals was constructed with 34 nodes and 99 edges (Figure 2). The statistical analysis from the network showed that GLY was connected with the most genes and signaling pathways (17 nodes and 16 edges). The top five gene targets assessed by the number of connections and degrees for the eight-compound network included MAPK14, MAPK8, NOS3, EGFR, and SRC. The top common pathway was the MAPK signaling pathway. It was noticed that the network clearly displayed a multitarget pattern of each phytochemical that has been associated with multiple genes and signaling pathways, and they also have crosstalk as reflected by the overlapping gene targets and signaling pathways.

### 3.2. Molecular Docking Analysis

**Figure 3:**
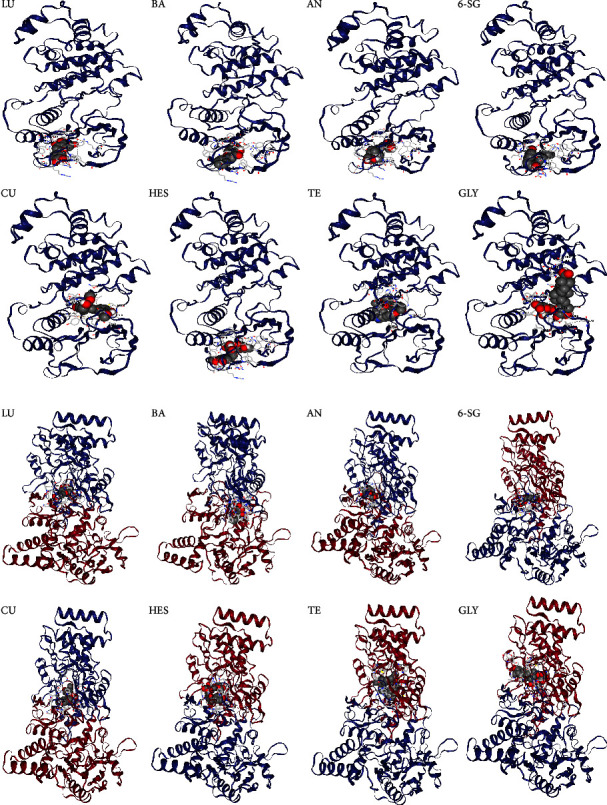
Molecular docking analysis of the eight phytochemical candidates with MAPK14 (a) and NOS3 (b) analysed by CB dock.

Based on the network pharmacology analysis, MAPK14 and NOS3 were the top two hub gene targets of all the eight phytochemicals. In order to verify this analysis, molecular docking was performed to evaluate the binding affinity between each phytochemical to MAPK14 and NOS3-coded proteins, respectively. The binding indices of each phytochemical on MAPK and NOS3-coded proteins are shown in Figures 3(a) and 3(b), respectively. The cavity size and affinity were evaluated using CB-Dock. The center represents the docking pocket center coordinates. The size parameters *x*, *y*, and *z* represent the directions of the docking pocket.

The relevant indices including affinity, cavity size, and binding location (center and size) for MAPK14 and NOS3 are shown in Tables 2 and 3, respectively. The binding energy < −5 kcal/mol was considered as the high affinity between the phytochemical and the target protein [56]. Herein, the binding affinities of the eight phytochemicals with MAPK14 and NOS3 were all less then −5, suggesting high affinities. Particularly, GLY and HES had stronger binding activities with MAPK14 (affinity <−10) than other phytochemicals, and TE showed the strongest binding with iNOS (affinity <−10).

### 3.3. Identification of Potential Synergy among the Eight Phytochemicals against Neuroinflammation

#### 3.3.1. NO Inhibitory Activity of Single Phytochemicals on LPS-Induced N11 Cells

The Alamar blue assay was conducted to examine the cytotoxicity of the eight phytochemicals on N11 cells. TE exhibited dose-dependent cytotoxicity from 10 to 100 *μ*M. HES, LU, and CU showed moderate cytotoxicity from 50 to 100 *μ*M. In contrast, AN, BA, 6-SG, and GLY did not induce any cytotoxicity from 0 to 100 *μ*M.

**Figure 4:**
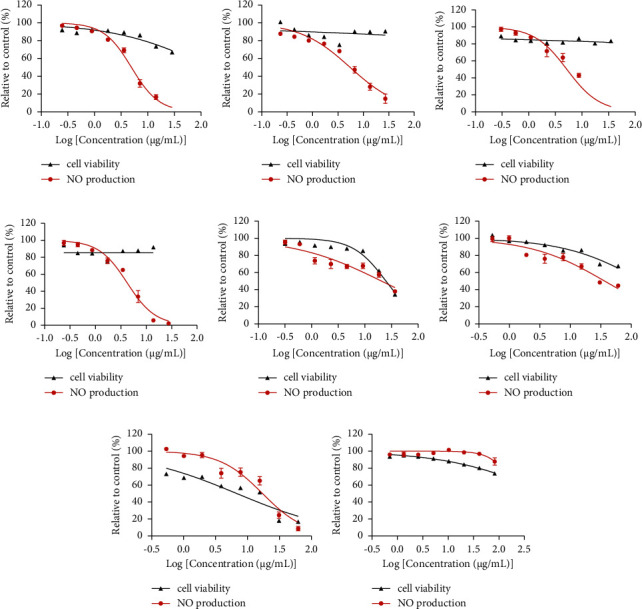
The NO inhibitory activities and cell viability of (a) LU, (b) BA, (c) AN, (d) 6-SG, (e) CU, (f) HES, (g) TE, and (h) GLY in LPS-activated N11 microglial cells. Data are shown as mean ± SEM (*n* > 3).

The cellular neuroinflammation model was established by activating the microglial N11 cells with LPS, which led to an excessive amount of NO to 24.48 ± 0.21 ng/mL (*p* < 0.0001*vs*. blank control: 0.41 ± 0.12 ng/mL). BA, AN, LU, and 6-SG lowered LPS-induced NO expression levels in a dose-dependent manner (Figure 4). Based on the dose-response curves, the IC_50_ values for each active phytochemical were calculated. 6-SG showed the highest potency (IC_50_ = 4.15 *μ*g/mL, 15.02 *μ*M), followed by LU (IC_50_ = 4.94 *μ*g/mL, 17.24 *μ*M), AN (IC_50_ = 5.22 *μ*g/mL, 14.88 *μ*M), and BA (IC_50_ = 5.49 *μ*g/mL, 20.30 *μ*M). CU and TE exhibited moderate NO inhibitory effects with IC_50_ values of 20.54 *μ*g/mL (34.59 *μ*M) and 17.25 *μ*g/mL (27.70 *μ*M), respectively. HES and GLY did not show any obvious inhibitory effects.

#### 3.3.2. Synergistic Effects of AN-SG and BA-SG Combinations on LPS-Induced NO Inhibition

The paired combinations of the eight phytochemicals were tested on LPS-induced N11 cells. Our results showed that AN-SG and BA-SG (0.50–31.34 *μ*g/mL) appeared to be more potent than their individuals, and the enhanced NO inhibitory activities were not associated with cytotoxicity. The rest of the pair-wised combinations did not show higher potencies than their individual components in general (Supplementary Material 3).

**Figure 5:**
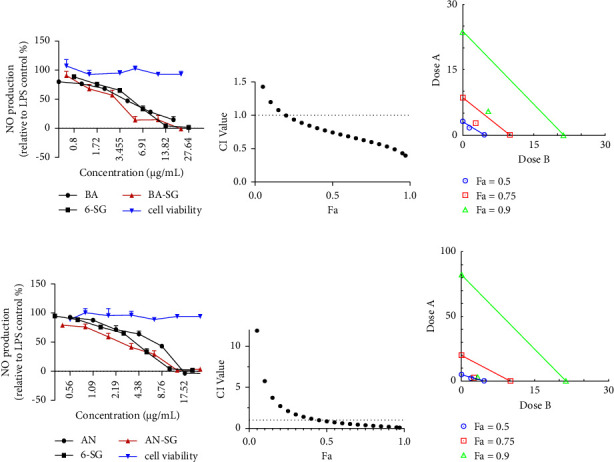
AN-SG and BA-SG combinations exhibited synergistic inhibitory effects on LPS-induced NO production in N11 cells. (a) AN, 6-SG, and AN-SG dose-dependently inhibited LPS-induced NO in N11 cells (*n* ≥ 3). (b) The synergistic NO inhibitory effect of AN-SG was determined by the CI-Fa curves. CI values represent the interaction in AN-SG, with CI < 1, CI = 1, and CI > 1 referring to synergy, addition, and antagonism, respectively. Fa on the *X*-axis is defined as the fraction effect level, and herein it refers to the NO inhibitory effect, respectively. (c) Isobologram analysis of AN-SG in NO inhibition when the default set of Fa values at 0.50, 0.75, and 0.9. (d) BA, 6-SG, and BA-SG dose-dependently inhibited LPS-induced NO in N11 cells (*n* ≥ 3). (e) The synergistic NO inhibitory effect of BA-SG was determined by the CI-Fa curves. CI values represent the interaction in BA-SG, with CI < 1, CI = 1, and CI > 1 referring to synergy, addition, and antagonism, respectively. Fa on the *X*-axis is defined as the fraction effect level, and herein it refers to the NO inhibitory effect, respectively. (f) Isobologram analysis of BA-SG in NO inhibition when the default set of Fa values at 0.50, 0.75, and 0.9.

AN-SG combination (Figure 5(a)) exhibited a dose-dependent NO inhibition with the IC_50_ value of 2.85 ± 0.66 *μ*g/mL, and it was significantly lower than that of AN (IC_50_ = 5.22 ± 0.91 *μ*g/mL) or 6-SG alone (IC_50_ = 4.16 ± 0.46 *μ*g/mL, both *p* < 0.0001). The CI model was then performed to evaluate the drug interaction responsible for the enhanced activity in the combination. The CI-Fa curve (Figure 5(b)) displayed a strong synergy of AN-SG combination in inhibiting NO, with CI values ranging from 0.39 to 0.99 when the Fa was above 0.20 (20%–97% NO inhibitory effect). The isobologram (Figure 5(c)) also supported the observed synergy of AN-SG in reducing LPS-stimulated NO when Fa values were at 0.50 (representing 50% of the NO inhibition).

Both BA and 6-SG showed a dose-dependent inhibition of NO, as shown in Figure 5(d). The BA-SG combination (IC_50_ = 3.28 ± 0.81 *μ*g/mL) exhibited a more prominent effect than that of BA (IC_50_ = 5.48 ± 0.83 *μ*g/mL) or 6-SG (IC_50_ = 4.16 ± 0.46 *μ*g/mL) alone (both *p* < 0.0001). The CI model revealed a synergistic interaction of BA and 6-SG when used together to suppress NO (Figure 5(e)). At the concentration range of 3.89–8.56 *μ*g/mL, a synergistic effect was observed with CI values ranging from 0.09 to 0.99 when the Fa was over 0.45 (45%–97% NO inhibitory effect). When Fa values were at 0.5 (representing 50% of the NO inhibition), the isobologram in Figure 5(f) further corroborated the reported synergy of BA-SG in decreasing LPS-stimulated NO.

#### 3.3.3. Compound-Gene Targets-Signaling Pathway Networks of AN-SG and BA-SG Combinations in Relation to Neuroinflammation

**Figure 6:**
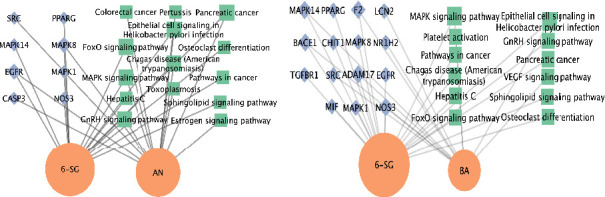
Compound-gene targets-signaling pathway networks for the AN-SG (a) and BA-SG (b). The green nodes represent the signaling pathway, the orange nodes represent the phytochemicals, and the blue nodes represent potential common compound targets in neuroinflammation. The size of each label represents its degree.

The compound-gene targets-signaling pathway networks of the AN-SG and BA-SG combinations were built to understand the associated mechanisms of their synergistic interaction. As shown in Figure 6, MAPK14 and NOS3 appeared to be the top hub gene targets, and the MAPK signalling pathway is the top overlapping pathway for both paired combinations. Then, the modulatory effects of these two combinations in comparison with their individual component on MAPK14 and iNOS protein expressions were examined by Western blot analysis.

#### 3.3.4. Synergistic Mechanisms of AN-SG and BA-SG Combinations in Relation to Modulated Phosphor-MAPKp38/MAPKp38 and iNOS Protein Expression Based on the Network Pharmacology Analysis

To verify the network pharmacology analysis and investigate the synergistic mechanism of AN-SG and BA-SG combinations, the protein levels of phosphor-MAPKp38/MAPKp38 (p-p38/p38) and iNOS were investigated.

**Figure 7:**
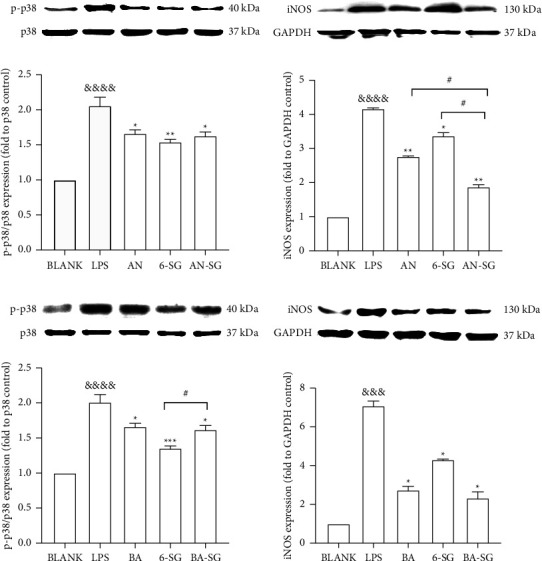
Cells were cultured in *T*75 cell flasks and were pretreated with AN, BA, 6-SG, AN-SG, and BA-SG 1 h prior to LPS (1 *μ*g/mL) for 0.5 h or 24 h protein expression levels of p-p38/p38 (a, c), iNOS (b, d) were analysed by western blot. All results (*n* = 3) are expressed as the mean ± SEM, ^&&&^*p* < 0.001, ^&&&&^*p* < 0.0001*vs*. BLANK, ^*∗*^*p* < 0.05, ^*∗∗*^*p* < 0.01, ^*∗∗∗*^*p* < 0.001, *vs*. LPS, ^#^*p* < 0.05*vs*. combination, by one-way ANOVA analysis with the Tukey test in GraphPad prism 9.

As shown in Figures 7(a) and 7(b), the stimulation of LPS (1 *μ*g/mL) led to significantly upregulated expressions of p-p38/p38 (*p* < 0.0001) and iNOS (*p* < 0.001) with fold increases of 2.06 ± 0.22 and 4.16 ± 0.08 in comparison to that of the untreated cells (Blank), respectively. AN, 6-SG, and AN-SG all significantly inhibited the increased fold change of p-p38/p38 (*p* < 0.05*vs*. LPS) and iNOS (*p* < 0.05*vs*. LPS). In addition, the inhibitory effect of the AN-SG combination was significantly greater than that of AN or 6-SG alone on iNOS (*p* < 0.05*vs*. AN or 6-SG).

The LPS stimulation (1 *μ*g/ml) caused elevated expressions of p-p38/p38 (*p* < 0.0001*vs*. Blank) and iNOS (*p* < 0.0001 vs. Blank) with fold increases of 2.01 ± 0.21 and 7.07 ± 0.49, respectively, in contrast to that of the untreated cells (Figures 7(c) and 7(d)). BA, 6-SG, and BA-SG all significantly downregulated the fold change of p − p38/p38 (*p* < 0.05 vs. LPS) and iNOS (*p* < 0.05 vs. LPS). It was noticed that the combined p-p38/p38 inhibitory effect was significantly higher than that of 6-SG alone.

## 4. Discussion

Network pharmacology analysis has emerged as a powerful tool for the development of combination therapy [57] and is particularly useful in Chinese herbal medicine research to understand the multitargeted mechanisms of the bioactive components in complex herbal formulations [58]. Herein, network pharmacology has been applied to evaluate the pharmacological actions of eight phytochemicals selected from Chinese herbs targeting neuroinflammation. Molecular docking and experimental bioassays were followed to explore synergistic interactions and the associated mechanisms of the pair-wised combinations among eight phytochemicals. Our results demonstrated that two paired combinations exhibited synergy in inhibiting LPS-induced NO production on microglia N11 cells, and the mechanisms were associated with the downregulation of MAPK p-p38/p38 and iNOS protein expressions. The data obtained from the experimental study is in line with the illustration from the network pharmacology and molecular docking analysis.

The highly relevant gene targets for the antineuroinflammatory effects of the eight phytochemicals included MAPK14 and MAPK8. The top KEGG pathway involved in the actions of the eight phytochemicals was the MAPK signaling pathway. MAPKs are a family of serine/threonine protein kinases that regulate key biological processes as well as cellular responses to external stress signals [59]. MAPKs are vital for intracellular signal transduction and play critical roles in regulating cell proliferation, brain plasticity, inflammatory responses, and other biological functions [60]. According to recent preclinical studies, increased MAPK activation is a significant factor in brain inflammation. p38*α*/MAPK14 and extracellular signal-regulated kinase (ERK) are intracellular signaling regulators [61, 62], which mediate the expression of the iNOS and TNF genes in LPS-activated glial cells, suggesting the role of p38MAPK in the activated glial cells [63, 64]. Increasing evidence showed that MAPK cascades, including mitogen- and stress-activated kinase 1 and mitogen- and stress-activated kinase 2, were associated with the production of IL-1 in the BV-2 mouse microglia cell line and primary rat microglia [65]. MAPK14 is ubiquitously expressed and plays an important role in proinflammatory signaling, making it an appealing therapeutic target for chronic inflammatory diseases [66]. Anti-neuroinflammatory therapies might be directed by targeting MAPKs kinases, such as MAPK p38 and their role in the transcription and translation of inflammation mediators, and can lead to an enhanced therapeutic outcome [67]. Our network pharmacology findings demonstrated that the MAPK signaling pathway was the common relevant pathway linking the actions of the phytochemicals to neuroinflammation, which correlates with previous findings.

Nitric oxide synthases (NOS) are the enzymes responsible for NO generation. To date, three distinct NOS isoforms have been identified, including neuronal NOS (nNOS/NOS1), inducible NOS (iNOS/NOS2), and endothelial NOS (eNOS/NOS3) [68]. The excessive NO is one of the important neuroinflammatory mediators that trigger neuronal toxicity and death [69]. A study by Connelly et al. [72] suggested that mice with macrophages NOS3 knock-out revealed downregulated nuclear factor kappa B (NF-*κ*B) signaling and reduced expression of iNOS, resulting in decreased LPS-induced NO generation and ultimately suppressed neuroinflammatory response [70]. Thus, these studies supported the results from our network pharmacology analysis that NOS3 is an important mediator in neuroinflammation. Moreover, iNOS was generated in activated microglial cells and mediated NO synthesis [71]. Studies in patients with Parkinson's disease revealed an increased density of iNOS-expressing glial cells in the substantia nigra compared to the control [72]. In addition, iNOS has been linked with microglial activation, inducing an inflammatory response and resulting in neural cell death [73]. Apart from the most relevant disease proteins and therapeutic targets discussed above, other neuroinflammation targets including EGFR, SRC, CASP3, and PPARG in the compound-gene targets-signaling pathway networks may potentially participate in the pharmacological actions of the eight phytochemicals against neuroinflammation.

Molecular docking is commonly used to model the interaction between small molecules and target proteins [74] and to estimate the binding energy of a ligand and the intensity of the interactions. Thus, molecular docking has the capacity to identify novel phytochemicals of therapeutic interest, predict the ligand-target interactions at a protein level, and determine the degree of binding affinity between a phytochemical and its target proteins [75]. Our results suggested that all eight phytochemicals exhibited strong binding affinity with MAPK14 and NOS3 according to intermolecular interactions. This result indicated that these phytochemicals displayed good binding activities with the hub targets of neuroinflammation, illustrating the mechanisms of action underlying the therapeutic effects of these phytochemicals [76]. Our molecular docking results provide evidence to support the network pharmacology findings at the molecular level.

*In vitro* bioassays were carried out to validate the results of the network pharmacology analysis and to identify possible synergistic interactions among the eight phytochemicals. LPS-induced microglia N11 cells were used to test the individual and combined activities of the eight phytochemicals in inhibiting NO production. Our results suggested that LU, AN, BA, and 6-SG lowered LPS-induced NO expression levels in a dose-dependent manner. Interestingly, although HES and GLY showed high binding affinities to both MAPK14 and NOS3 proteins, they did not show any obvious inhibitory effect in the NO assay, which may be attributed to their broad and unspecific effects. Furthermore, our study demonstrated that the AN-SG and BA-SG combinations exhibited synergistic interaction in reducing NO production. To the best of our knowledge, it is the first study demonstrating synergistic interactions between AN and 6-SG and BA and 6-SG targeting neuroinflammation. Based on the constructed network, the MAPK14 and NOS3 gene targets were highly relevant for the anti-neuroinflammatory actions of the AN-SG and BA-SG combinations, both linking to the downstream production of iNOS protein expression [77, 78]. Our Western blot analysis suggested that the synergistic mechanisms may be associated with downregulated phosphor-MAPKp38 and iNOS proteins. These findings are in line with the predictions from our network pharmacology analysis that the crosstalk among AN, 6-SG, and BA were likely to occur on their hub gene targets, MAPK8, MAPK14 and NOS3, and their coded proteins.

Previous studies suggested that AN, BA, and 6-SG exhibited anti-neuroinflammatory activity *via* the downregulation of NF-*κ*B and MAPK signalling pathways. AN downregulated the protein levels of iNOS and cyclooxygenase-2 (COX-2) and mRNA expression in LPS-induced human keratinocyte HaCaT cells [79]. BA significantly inhibited the activation of MAPKs and suppressed the transcriptional activity of NF-*κ*B [80]. Similarly, treatment with 6-SG resulted in the reduction of LPS-induced NF-*κ*B subunit and the dependent transcriptional activity of NF-*κ*B by blocking the phosphorylation of NF-kappa-B inhibitor alpha(I*κ*B*α*) and subsequent degradation of I*κ*B*α* [81]. 6-SG also interferes with the activation of PI3K/Akt/I*κ*B kinases (IKK) and MAPK [20]. Thus, the common signaling pathways of NF-*κ*B and MAPK may be the key to the synergistic mechanism of the AN-SG and BA-SG combinations.

The current study only focused on analysing the MAPK pathway based on the network pharmacology results. More in-depth studies of other hub KEGG pathways (i.e. NF-*κ*B) and their target proteins by the two combinations will be conducted in the future. Moreover, it is evident that AN, 6-SG, and BA were associated with many other key gene targets and coded proteins, as displayed by the compound-gene target network. These gene targets and signalling pathways may also contribute to the synergistic effects of AN-SG and BA-SG against neuroinflammation.

The network pharmacology replies primarily to the currently available data from online databases, which may lead to a biased result [82]. Therefore, it should only be used as a predictive tool for the molecular mechanisms of a compound mixture and reveal the relevant relationship between the phytochemical and gene targets without providing further information on their downregulatory or upregulatory actions. To elucidate the detailed mode of action, it is no doubt that more experimental investigations should be carried out. We also recognise that the cellular assays used in this study serve as a primary screening method. Whether the AN-SG and BA-SG can be applied to a broader range of neuroinflammatory mediators and reserve the synergistic actions in a whole organism and a clinical setting are yet to be determined. Also, it is unclear if synergy occurs in the high-order combinations among eight phytochemicals which requires further investigation.

## 5. Conclusions

The present study developed two novel herbal compound combinations, AN-SG and BA-SG, that showed synergistic anti-neuroinflammatory activities. The associated mechanisms underlying the observed synergy were explored through network pharmacology analysis and molecular docking. Network pharmacology demonstrated that MAPK14, MAPK8, and NOS3 were the main gene targets of AN, 6-SG, and BA, and the top KEGG pathway was the MAPK signaling pathway. Western blot analysis demonstrated that AN-SG and BA-SG showed prominent effects in inhibiting p-p38 MAPK and iNOS, which has partly validated the illustration from network pharmacology. The present study provides insight into the development of synergistic combinations targeting neuroinflammation with integrated network pharmacology, molecular docking, and experimental bioassays.

## Figures and Tables

**Table 1:** Characteristic pharmacological actions of eight phytochemicals with potential anti-neuroinflammatory activity.

Phytochemicals	Molecular formula	Molecular weight (g/mol)	OB	DL	BBB	Number of the total gene targets	Hub gene targets with neuroinflammation
LU	C_15_H_10_O_6_	286.24	36.16	0.25	−0.84	86	8
BA	C_15_H_10_O_5_	270.24	33.52	0.21	−0.05	49	8
AN	C_20_H_30_O_5_	350.45	53.44	0.35	−0.94	57	10
6-SG	C_17_H_24_O_3_	276.37	31.00	0.14	0.49	47	12
CU	C_21_H_20_O_6_	368.38	5.15	0.41	−0.76	47	9
HES	C_28_H_34_O_15_	610.18	13.33	0.67	−2.70	83	14
TE	C_38_H_42_N_2_O_6_	622.70	26.64	0.10	0.44	46	10
GLY	C_42_H_62_O_16_	822.90	19.62	0.11	−2.86	90	24

**Table 2:** Binding indices of the eight phytochemicals with MAPK14 analysed by molecular docking.

Chemicals	Affinity (kcal/mol)	Cavity size	Center (*x*, *y*, *z*)	Size (*x*, *y*, *z*)
LU	−9.80	12601	18, 9, 37	34, 34, 35
BA	−9.20	12601	18, 9, 37	34, 34, 35
AN	−8.60	12601	18, 9, 37	34, 34, 35
6-SG	−7.40	12601	18, 9, 37	34, 34, 34
CU	−8.80	12601	18, 9, 37	34, 34, 35
HES	−10.10	12601	18, 9, 37	34, 34, 35
TE	−9.60	574	34, 9, 22	23, 23, 23
GLY	−10.10	574	34, 9, 22	30, 30, 30

**Table 3:** Binding indices of the eight phytochemicals with NOS3 analysed by molecular docking.

Chemicals	Affinity (kcal/mol)	Cavity size	Center (*x*, *y*, *z*)	Size (*x*, *y*, *z*)
LU	−9.40	12459	17, 9, 40	35, 35, 35
BA	−9.50	12459	17, 9, 40	35, 35, 35
AN	−8.10	12459	17, 9, 40	35, 35, 35
6-SG	−7.70	12459	17, 9, 40	35, 35, 35
CU	−9.00	12459	17, 9, 40	35, 35, 35
HES	−10.20	12459	17, 9, 40	35, 35, 35
TE	−10.30	12459	17, 9, 40	35, 35, 35
GLY	−10.20	12459	17, 9, 40	35, 35, 35

## Data Availability

The analysed data used to support the findings of this study are included within the article, and the raw data used to support the findings of this study are available from the corresponding author upon request.

## Supplementary Materials

Supplementary Materials Supplementary Material 1: HPLC analysis of the isolated phytochemicals used in the study (Chengdu BioPurify Pty Co., China). (A) LU purity: 98.62%, retention time: 10.29 min. (B) BA purity: 98.63%, retention time: 13.65 min, (C) AN purity: 99.33%, retention time: 12.55 min, (D) 6-SG purity: 98.70%, retention time: 13.67 min, (E) CU purity: 99.95%, retention time: 11.42 min, (F) HES purity: 99.20%, retention time: 9.84 min, (G) TE purity: 99.20%, retention time: 10.16 min, and (H) GLY purity: 99.70%, retention time: 9.82 min. Supplementary Material 2: Venn diagram of the number of relevant gene targets of eight phytochemicals and neuroinflammation. Supplementary Material 3: The PPI interaction network for eight phytochemicals related to neuroinflammation. The nodes in the figure represent proteins, and the edges represent the interrelationships between proteins. Supplementary Material 4: GO enrichment analysis of BP, CC, and MF for eight phytochemicals related to neuroinflammation. Supplementary Material 5: KEGG pathway analysis of potential targets in eight phytochemicals. The size of the bubbles refers to the gene counts of the phytochemical and the scale of colours refer to the *p* values from large to small. Up to top 20 KEGG pathways are shown for each phytochemical which were determined by *p* values. Supplementary Material 6: MAPK signaling pathway map constructed by the KEGG mapper (KEGG PATHWAY: MAPK signaling pathway—*Homo sapiens* (human) (genome.jp)) [83]. Supplementary Material 7: The dose-response curves of paired combinations and their corresponding component of eight phytochemicals that dose-dependently inhibited NO and cell viability of in LPS-induced N11 cells (*n* ≥ 3).
