# Supplementary material for: Development of Novel Herbal Compound Formulations Targeting Neuroinflammation: Network Pharmacology, Molecular Docking, and Experimental Verification
Source: Evid Based Complement Alternat Med. 2023 May 24;2023:2558415. doi: 10.1155/2023/2558415 (PMC10232107; doi:10.1155/2023/2558415)
Supplement: Supplementary Materials — Supplementary Material 1: HPLC analysis of the isolated phytochemicals used in the study (Chengdu BioPurify Pty Co., China). (A) LU purity: 98.62%, retention time: 10.29 min. (B) BA purity: 98.63%, retention time: 13.65 min, (C) AN purity: 99.33%, retention time: 12.55 min, (D) 6-SG purity: 98.70%, retention time: 13.67 min, (E) CU purity: 99.95%, retention time: 11.42 min, (F) HES purity: 99.20%, retention time: 9.84 min, (G) TE purity: 99.20%, retention time: 10.16 min, and (H) GLY purity: 99.70%, retention time: 9.82 min. Supplementary Material 2: Venn diagram of the number of relevant gene targets of eight phytochemicals and neuroinflammation. Supplementary Material 3: The PPI interaction network for eight phytochemicals related to neuroinflammation. The nodes in the figure represent proteins, and the edges represent the interrelationships between proteins. Supplementary Material 4: GO enrichment analysis of BP, CC, and MF for eight phytochemicals related to neuroinflammation. Supplementary Material 5: KEGG pathway analysis of potential targets in eight phytochemicals. The size of the bubbles refers to the gene counts of the phytochemical and the scale of colours refer to the p values from large to small. Up to top 20 KEGG pathways are shown for each phytochemical which were determined by p values. Supplementary Material 6: MAPK signaling pathway map constructed by the KEGG mapper (KEGG PATHWAY: MAPK signaling pathway—Homo sapiens (human) (genome.jp)) [83]. Supplementary Material 7: The dose-response curves of paired combinations and their corresponding component of eight phytochemicals that dose-dependently inhibited NO and cell viability of in LPS-induced N11 cells (n ≥ 3). [file 2558415.f1.zip › Supplementary 1-1.pdf]

A

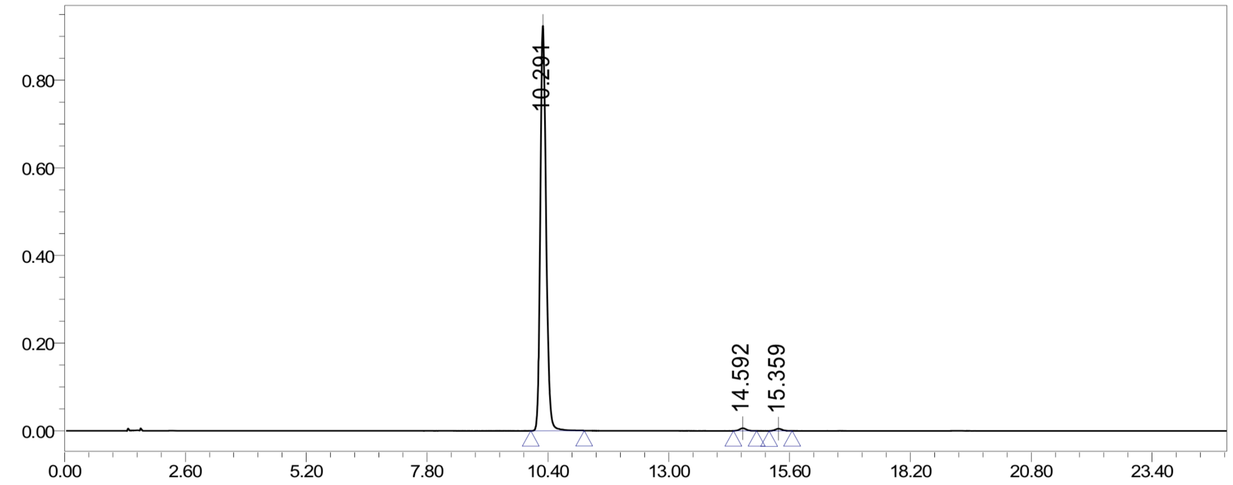

Peaks	Retention (min)	Peak area (mAU*s)	Peak height (μV)	Peak are (%)
1	10.291	8037396	98.62	924369
2	14.592	61611	0.76	6045
3	15.359	51237	0.63	4986

B

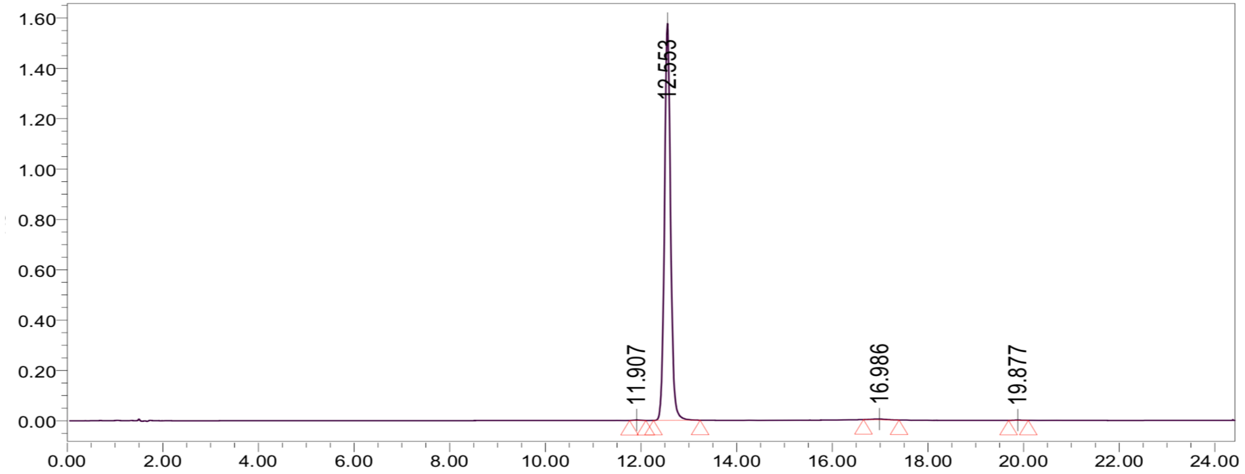

Peaks	Retention (min)	Peak area (mAU*s)	Peak height (μV)	Peak are (%)
1	11.907	15070		0.11
2	12.553	14016843		99.33
3	16.986	65681		0.47
4	19.877	14248		0.1

C

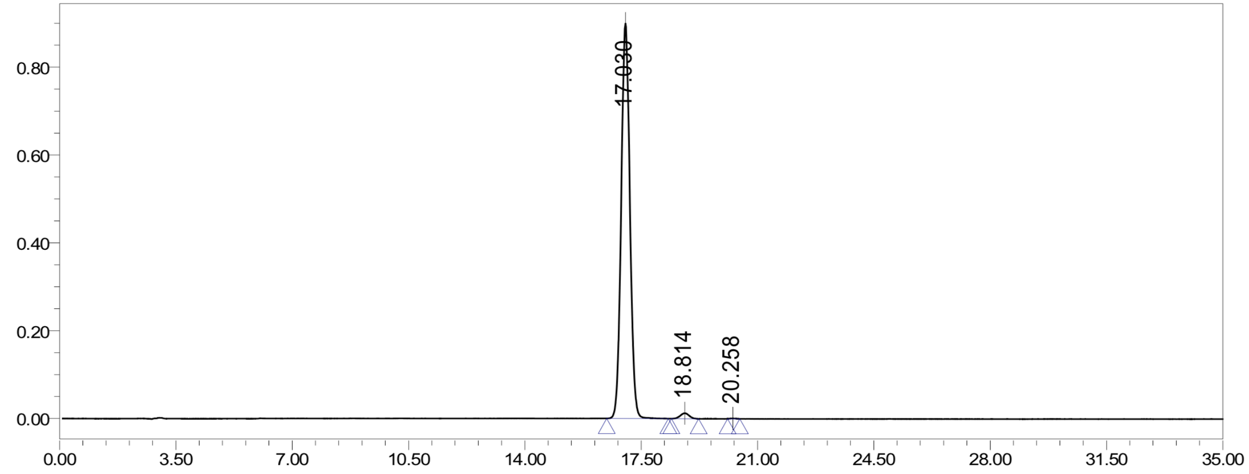

Peaks	Retention (min)	Peak area (mAU*s)	Peak height (μV)	Peak are (%)
1	4.732	9788	0.09	1624
2	5.898	22093	0.21	3453
3	12.981	13427	0.13	984
4	13.651	10351271	98.63	105299 2
5	21.685	8895	0.08	620
6	22.593	15742	0.15	1076
7	24.334	73520	0.7	4204
