# Supplementary material for: Development of Novel Herbal Compound Formulations Targeting Neuroinflammation: Network Pharmacology, Molecular Docking, and Experimental Verification
Source: Evid Based Complement Alternat Med. 2023 May 24;2023:2558415. doi: 10.1155/2023/2558415 (PMC10232107; doi:10.1155/2023/2558415)
Supplement: Supplementary Materials — Supplementary Material 1: HPLC analysis of the isolated phytochemicals used in the study (Chengdu BioPurify Pty Co., China). (A) LU purity: 98.62%, retention time: 10.29 min. (B) BA purity: 98.63%, retention time: 13.65 min, (C) AN purity: 99.33%, retention time: 12.55 min, (D) 6-SG purity: 98.70%, retention time: 13.67 min, (E) CU purity: 99.95%, retention time: 11.42 min, (F) HES purity: 99.20%, retention time: 9.84 min, (G) TE purity: 99.20%, retention time: 10.16 min, and (H) GLY purity: 99.70%, retention time: 9.82 min. Supplementary Material 2: Venn diagram of the number of relevant gene targets of eight phytochemicals and neuroinflammation. Supplementary Material 3: The PPI interaction network for eight phytochemicals related to neuroinflammation. The nodes in the figure represent proteins, and the edges represent the interrelationships between proteins. Supplementary Material 4: GO enrichment analysis of BP, CC, and MF for eight phytochemicals related to neuroinflammation. Supplementary Material 5: KEGG pathway analysis of potential targets in eight phytochemicals. The size of the bubbles refers to the gene counts of the phytochemical and the scale of colours refer to the p values from large to small. Up to top 20 KEGG pathways are shown for each phytochemical which were determined by p values. Supplementary Material 6: MAPK signaling pathway map constructed by the KEGG mapper (KEGG PATHWAY: MAPK signaling pathway—Homo sapiens (human) (genome.jp)) [83]. Supplementary Material 7: The dose-response curves of paired combinations and their corresponding component of eight phytochemicals that dose-dependently inhibited NO and cell viability of in LPS-induced N11 cells (n ≥ 3). [file 2558415.f1.zip › Supplementary 1-2.pdf]

D

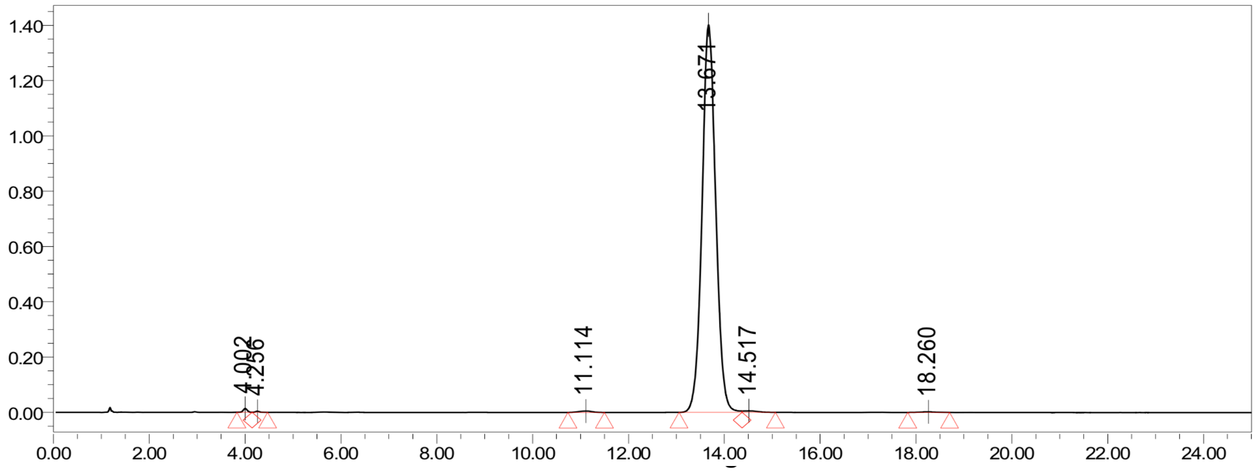

Peaks	Retention (min)	Peak area (mAU*s)	Peak height (µV)	Peak are (%)
1	4.002	84421	14096	0.3
2	4.256	25213	0.09	3935
3	11.114	96920	0.34	5031
4	13.671	27854508	98.7	1401299
5	14.517	108216	0.38	5293
6	18.26	52082	0.18	2143

E

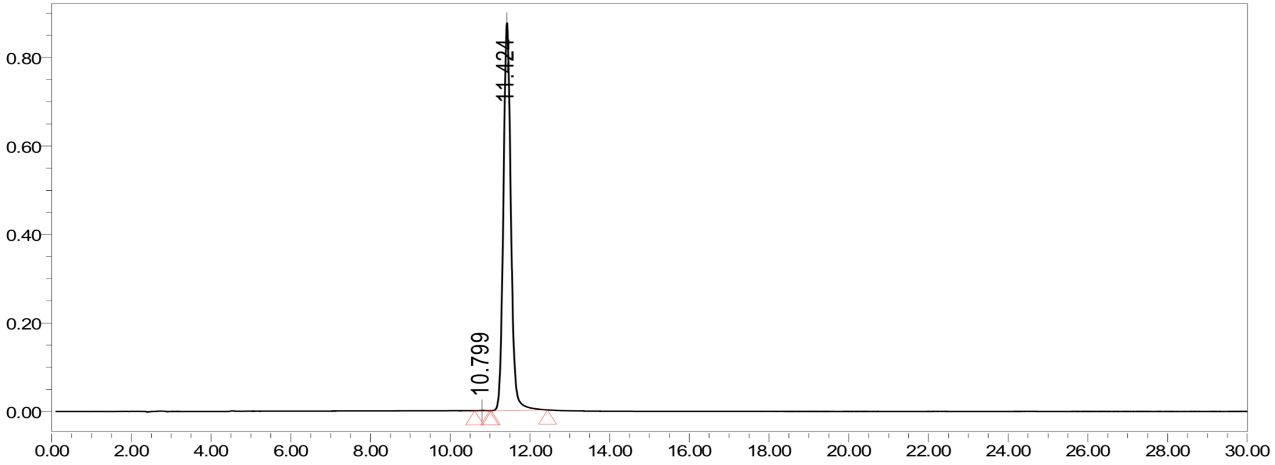

Peaks	Retention (min)	Peak area (mAU*s)	Peak height (µV)	Peak are (%)
1	10.799	5255	0.05	513
2	11.424	11500817	99.95	875514

F

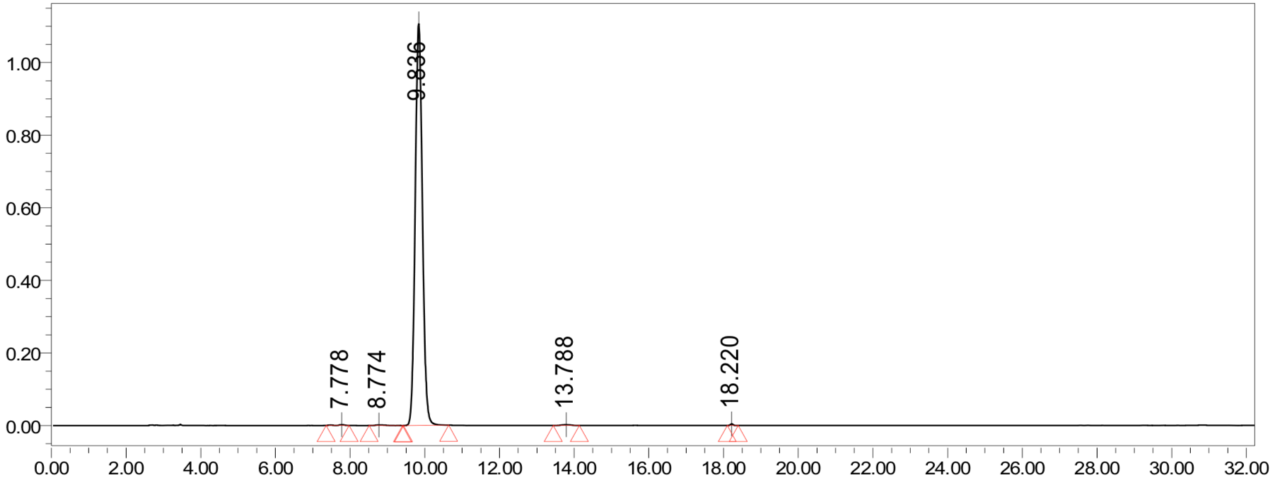

Peaks	Retention (min)	Peak area (mAU*s)	Peak height (µV)	Peak are (%)
1	7.778	28443	0.19	2175
2	8.774	30987	0.21	1737
3	9.836	14827095	99.2	110675 7
4	13.788	39748	0.27	2262
5	18.22	21073	0.14	4390
